# Facing the Omicron variant—how well do vaccines protect against mild and severe COVID-19? Third interim analysis of a living systematic review

**DOI:** 10.3389/fimmu.2022.940562

**Published:** 2022-08-24

**Authors:** Wiebe Külper-Schiek, Vanessa Piechotta, Antonia Pilic, Madeleine Batke, Léa-Sophie Dreveton, Brogan Geurts, Judith Koch, Stefan Köppe, Marina Treskova, Sabine Vygen-Bonnet, Maria Waize, Ole Wichmann, Thomas Harder

**Affiliations:** ^1^ Department of Infectious Disease Epidemiology, Robert Koch Institute, Berlin, Germany; ^2^ Centre for International Health Protection, Robert Koch Institute, Berlin, Germany; ^3^ Institute of Global Health, Heidelberg University Hospital, Heidelberg, Germany

**Keywords:** SARS-CoV-2, systematic review, vaccine effectiveness, vaccination, COVID-19, Omicron variant, variant of concern

## Abstract

**Background:**

The severe acute respiratory syndrome coronavirus 2 (SARS-CoV-2) Omicron variant is currently the dominant variant globally. This third interim analysis of a living systematic review summarizes evidence on the effectiveness of the coronavirus disease 2019 (COVID-19) vaccine (vaccine effectiveness, VE) and duration of protection against Omicron.

**Methods:**

We systematically searched literature on COVID-19 for controlled studies, evaluating the effectiveness of COVID-19 vaccines approved in the European Union up to 14/01/2022, complemented by hand searches of websites and metasearch engines up to 11/02/2022. We considered the following comparisons: full primary immunization vs. no vaccination, booster immunization vs. no vaccination, and booster vs. full primary immunization. VE against any confirmed SARS-CoV-2 infection, symptomatic, and severe COVID-19 (i.e., COVID-19-related hospitalization, ICU admission, or death) was indicated, providing estimate ranges. Meta-analysis was not performed due to high study heterogeneity. The risk of bias was assessed with ROBINS-I, and the certainty of the evidence was evaluated using GRADE.

**Results:**

We identified 26 studies, including 430 to 2.2 million participants, which evaluated VE estimates against infections with the SARS-CoV-2 Omicron variant. VE against any confirmed SARS-CoV-2 infection ranged between 0–62% after full primary immunization and between 34–66% after a booster dose compared to no vaccination. VE range for booster vs. full primary immunization was 34–54.6%. After full primary immunization VE against symptomatic COVID-19 ranged between 6-76%. After booster immunization VE ranged between 3-84% compared to no vaccination and between 56-69% compared to full primary immunization. VE against severe COVID-19 ranged between 3-84% after full primary immunization and between 12-100% after booster immunization compared to no vaccination, and 100% (95% CI 71.4-100) compared to full primary immunization (data from only one study). VE was characterized by a moderate to strong decline within 3–6 months for SARS-CoV-2 infections and symptomatic COVID-19. Against severe COVID-19, protection remained robust for at least up to 6 months. Waning immunity was more profound after primary than booster immunization. The risk of bias was moderate to critical across studies and outcomes. GRADE certainty was very low for all outcomes.

**Conclusions:**

Under the Omicron variant, the effectiveness of EU-licensed COVID-19 vaccines in preventing any SARS-CoV-2 infection is low and only short-lasting after full primary immunization, but can be improved by booster vaccination. VE against severe COVID-19 remains high and is long-lasting, especially after receiving the booster vaccination.

## Introduction

The Omicron variant of severe acute respiratory syndrome coronavirus 2 (SARS-CoV-2) [Phylogenetic Assignment of Named Global Outbreak (Pango) lineage designation B.1.1.529] was first detected in South Africa in November 2021. Since then, the variant spread rapidly across countries and has largely replaced all other variants globally [>99.8% of all sequences submitted to the Global Initiative on Sharing All Influenza Data (GISAID) were Omicron in week 5 of 2022] (1). Evidence suggests that the Omicron variant has a growth rate advantage compared to the previously dominant Delta variant, leading to its overtake as the dominant variant globally, while the Delta variant now only represents 0.1% of the collected samples (1).

High rates of asymptomatic infection and symptomatic COVID-19 disease among people previously infected with SARS-CoV-2 or fully vaccinated with a COVID-19 vaccine raise concerns that the currently available vaccines are less or no longer effective against the Omicron variant. To summarize the existing evidence on the effectiveness and the duration of protection conferred by COVID-19 vaccines licensed in the European Union (EU) with respect to the Omicron variant and compare it to the Delta variant, we synthesized the evidence within an ongoing living systematic review (LSR), conducted by the Robert Koch Institute (RKI) in collaboration with the National Immunisation Technical Advisory Groups (NITAGs) network coordinated by the European Centre for Disease Prevention and Control (ECDC) (2).

## Methods

### Literature search

The LSR follows the Preferred Reporting Items for Systematic Review and Meta-analysis (PRISMA) guidelines (**Supplementary Material 1**, Part 1) and was registered in the Prospective Register of Systematic Reviews (PROSPERO registration ID no. CRD42020208935; updated on 9 March 2022). All amendments since the initial registration are available online. The methods have been previously described in detail (2). In brief, we included studies of any design that had a comparison group and investigated vaccine effectiveness (VE) against SARS-CoV-2 infection of any severity of COVID-19 vaccines approved by the European Medicine Agency (EMA) in people ≥12 years of age (see **Supplementary Material 1**, Part 2, for complete PICO question). For this third update of the LSR, we only considered studies that reported on outcomes that are due to the SARS-CoV-2 Omicron variant or that occurred during a dominant circulation of the Omicron variant. There were no restrictions with regard to publication language or status.

We searched the COVID-19 literature database constructed by the RKI library (see **Supplementary Material 1**, Part 3, for the description of the database and the search strategy) for studies published between 23 October 2021 and 14 January 2022. We added literature identified by a hand search of websites and metasearch engines, indicated in **Supplementary Material 1**, Part 3, up to 11 February 2022. Potentially relevant publications were screened by examination of the title/abstract and full-text level by pairs of independent investigators (AP, SK, MB, LSD, VP, and/or WKS).

### Data extraction 

Data from the included studies (see PROSPERO registration for details) were extracted in duplicate and summarized in tables (AP, MB, VP, and WKS). We considered VE data on the following three comparisons by vaccine type (mRNA-based, vector-based, heterologous scheme, any vaccine): (1) full primary immunization vs. no vaccination (i.e., placebo, no vaccination, or vaccine not directed against COVID-19), (2) booster immunization vs. no vaccination, and (3) booster immunization vs. full primary immunization. We used the term “full primary immunization” to indicate a completed primary vaccination series, as defined by the study itself. Booster immunization indicates the vaccine dose following the full primary immunization. Outcomes of interest were VE against polymerase chain reaction- (PCR) or antigen-test confirmed SARS-CoV-2 infection of “any type” (i.e., studies did not indicate underlying symptoms), “symptomatic COVID-19,” and “severe COVID-19” (including hospitalization, ICU admission, or death due to SARS-CoV-2 infection). To investigate VE at different time points since vaccination, data were stratified into four time periods (≈14 days, >14 days up to 3 months, >3 months up to 6 months, and >6 months).

### Data synthesis

As there was substantial heterogeneity across studies, we abstained from conducting a meta-analysis but summarized the studies as follows: we assessed the range of VE of any mRNA- or vector-based vaccines or for heterologous schedules against the outcomes described for the different time strata by including VE estimates from all studies that provide VE estimates for the respective time point (**Table 1**). If studies reported VE data for more granular time points within the defined time stratum, it was always the estimate of the latest time point within the stratum that contributed to the indicated range (e.g., if studies reported VE after 2–4 and 5–9 weeks after vaccination, the estimate of 5–9 weeks was included in the depicted effect range). VE estimates closest to 14 days were included only for the time stratum of “≈14 days”. The latter stratum included further VE data that were only assessed at “≥14 days” after full primary vaccination, “≥7 days” post-booster vaccination, or when the time point of assessment after full primary immunization was not reported at all. To visualize VE over time after full primary and booster vaccination, the estimates contributing to the VE range for the respective time category were included in forest plots. Additionally, we assessed the percentage difference of VE over time in studies reporting VE estimates for at least two different time points. We provide the range of observed minimal and maximal differences across studies. For more detailed information, please refer to the table of all extracted information provided in **Supplementary Material 2**.

### Risk of bias and quality assessments

ROBINS-I was used to assess the risk of bias (26). The certainty of the evidence included in the LSR was rated using the Grading of Recommendations Assessment, Development, and Evaluation (GRADE) approach (27, 28).

**Table 1:** Effectiveness of COVID-19 vaccines against SARS-CoV-2 Omicron variant infection (infection (any type), symptomatic COVID-19, and severe COVID-19 (hospitalization, ICU admission, or death)).

	Vaccine(s) used for immunization	Range of adjusted vaccine effectiveness (95% CI)^a,b,c^			
		≈14 days^d^	>14 days up to 3 months	>3 months up to 6 months	>6 months
Infection (any type)					
**Full primary immunization**	mRNA-based (any)^e^	16% (0–37) to 55.2% (23.5–73.7); (3–8)	4.2% (-30.8–29.8) to 42.8% (33.8–50.7); (3, 4, 7)	-76.5% (-95.3– -59.5) to 23% (15.8–29.6); (3, 4)	8.6% (3.3–13.6); (4)
	Vector-based (any)^f^	-4% (-97–43) to 11.4 (NR to NR); (6, 7)	11.4% (NR to NR); (7)	..	..
	Any vaccine^h^	-13% (-38–8) to 62% (58–66); (9–11)	..	-38% (-61– -18); (10)	-16% (-62–17); (10)
	Heterologous scheme^g^	..	..	..	..
**Booster immunization**	mRNA-based (any)^e^	34% (16–49) to 66% (36–81); (4–6, 8, 10, 12)^i^	54.6% (30.4–70.4) ^i^; (3)	..	..
	Vector-based (any)^f^	..	..	..	..
	Any vaccine^h^	38% (29–46) to 76% (72–79); (6, 7, 9) ^i^	..	..	..
	Heterologous scheme^j^	..	..	..	..
**Symptomatic COVID-19**					
**Full primary immunization**	mRNA-based (any)^e^	41% (-57–77) to 76% (72–79); (13–15)	44.8% (16–63.8) to 54% (49–58); (13, 14)	13.3% (12.0–14.7) to 20.8% (13.7–27.4); (13, 14)	-9.4% (-16.3–2.8) to 13% (3–22); (13, 14)
	Vector-based (any)^f^	6% (-103–56) to 49.8% (40.7–57.5); (13, 15)	35.7% (27.7–42.8); (13)	6.1% (4.1–8.1); (13)	-1.0% (-2.4–0.3); (13)
	Any vaccine^h^	36% (24-45); (10)^k^	12% (3-21); (10)^k^	15% (8 – 22); (10)^k^	2% (-17 – 17); (10)^k^
	Heterologous scheme^g^	..	..	..	..
**Booster immunization**	mRNA-based (any)^e^	50.0% (41.2–57.4) to 73.9% (73.2–74.5); (12–14, 16)^i^	43.7% (32.9–52.7) to 65.4% (63.9–66.9); (13, 14)	..	..
	Vector-based (any)^f^	19% (-43–54); (15)	..	..	..
	Any vaccine^h^	61% (56-65); (10)^k^	..	..	..
	Heterologous scheme^j^	63.2% (62.6–63.8) to 70.7% (70.1–71.2); (13)	54.0% (53.3–54.8) to 62.1% (61.1–63.1); (13)	..	..
**Severe COVID-19 (hospitalization, ICU admission, or death)**					
**Full primary immunization**	mRNA-based (any)^e^	3% (-114–56) to 81% (65–90); (4, 13, 14, 17–22)	44% (-14–72) to 95% (57–99); (13, 22)	57.3% (42.7–68.2) to 91% (31–99); (13, 22)	34.9% (17.7–48.4) to 80.7% (71.3–87); (13, 14, 18, 20, 22)
	Vector-based (any)^f^	17% (-246–80) to 84% (-16–98); (19, 22)	21% (-81–66) to 85% (54–95); (22, 24)	-8% (-213–62) to 55.8% (34.1–70.3); (13, 22)	32.7% (19.7–43.6); (13)
	Any vaccine^h^	55% (-106–90) to 77% (-91–97); (6, 10, 23)	37% (-71 – 77); (10)	75% (51-87); (10)	41 % (-22–72) to 86% (-12 – 98); (10, 23)
	Heterologous scheme^g^	..	..	..	..
**Booster immunization**	mRNA-based (any)^e^	12% (-45–46) to 100% (71.4–100); (4, 10, 12–14, 18–22)^i^	78% (76–80) to 93.7% (80.3–98); (13, 20, 22)	..	..
	Vector-based (any)^f^	78% (76–80) to 84% (67–92); (22, 24)	79% (76–81) to 84% (80–88); (22)	..	..
	Any vaccine^h^	60% (-163–90) to 956% (87-98); (6, 10, 23)	..	..	..
	Heterologous scheme^j^	86.9% (82.8–90.1) to 91.4% (86.8–94.4); (13)	85% (81.2–88) to 91.2% (82.8–95.5); (13)	..	..

a If not indicated otherwise, vaccine effectiveness (VE) estimates refer to the comparison of vaccinated (2 or 3 doses, respectively) vs. unvaccinated;

b Provided VE estimates refer to the last reported time point per observation period (e.g. if studies reported VE after 2-4 and 5-9 weeks, the estimate of 5-9 weeks was included in the depicted effect range)

c Several effect ranges are derived from single studies providing data for different vaccine types

d time point closest to 14 days

e Comirnaty or Spikevax

f Vaxzevria or COVID-19-vaccine Janssen

g 1^st^ dose with vector-based vaccine followed by prime booster dose of mRNA-based vaccine

h Studies include recipients of different vaccine types, and data was not further stratified

i Includes at least one study comparing booster vs. primary vaccination schedules (i.e., 3 vs. 2 doses)

j 1^st^ dose with vector-based vaccine followed by prime booster dose, and 3^rd^ dose of mRNA-based vaccine OR vector-based primary vaccination followed by one booster dose of mRNA-based vaccine

k One study estimated VE against vaccinated individuals who received second dose before ≥25 weeks, because of insufficient unvaccinated individuals available for analysis (25). VE against symptomatic infection with Omicron and Delta was estimated for 16-49 and 50+ year old’s, respectively. Estimates are provided for all reported observation periods in **Supplementary Material 2**.

## Results

### Study Screening

**Figure 1:**
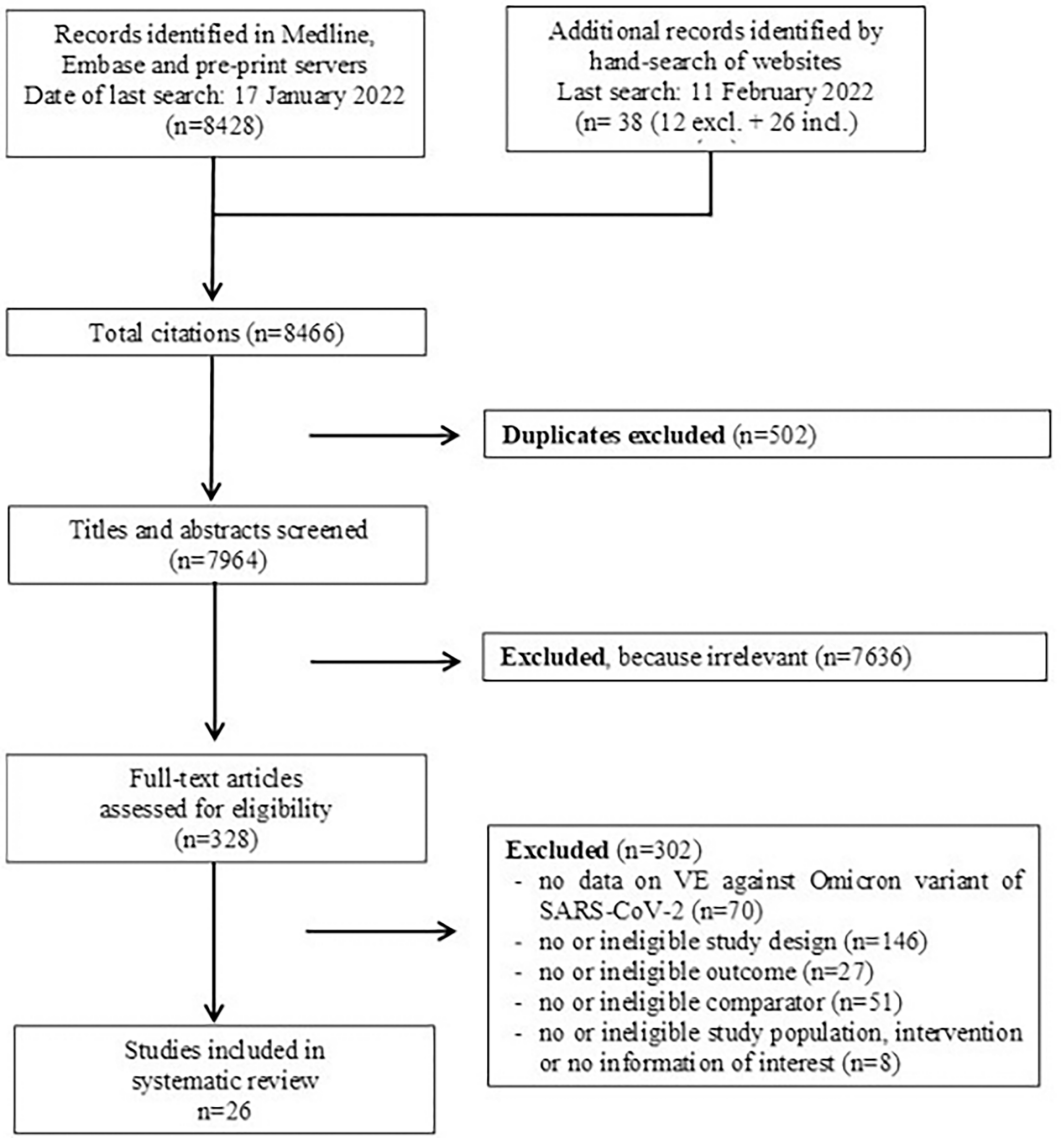
PRISMA flow diagram.

We identified a total of 8,428 entries in the database, until 14 January 2022. Another 38 potentially relevant studies were added by hand search up to 11 February 2022. After title/abstract and full-text screening, data from 26 studies (3–25, 29–31) were extracted (**Supplementary Material 1**, Part 4, **Figure 1** **)**. If studies referred to previously published references for further information, these references were considered and information extracted if necessary. For pre-print studies with several versions available, the most recent update published until 11 February 2022 was included.

### Characteristics of included studies

The included studies, of which most (21/26) were not yet peer-reviewed, reported VE estimates against infections (and related outcomes) with only the Omicron variant (5/26) or included VE data against infections with the Delta variant for comparison (21/26). Fourteen studies assessed VE using a test-negative design, eight based on a cohort design, three on a case–control design, and one study reported infection rates per vaccination status within a transmission study.

In the studies, the SARS-CoV-2 variant causing the investigated outcome was identified either from time periods with a known dominant circulation of the Omicron (resp. Delta) variant in the corresponding study location, from whole-genome sequencing (WGS), or through S-gene target failure (SGTF) in PCR assays. Studies using the latter method subdivided the SARS-CoV-2 positive samples by the detection or non-detection of the S-gene in a three gene PCR assay. As the SGTF is characteristic for the Omicron variant but rare in the Delta variant, studies used the detection of the S-gene as a proxy for the Delta variant and the “non-detection” as a proxy for the Omicron variant. As most studies report overall VE estimates without further differentiating between WGS- or SGTF-variant assessment, the data were synthesized only into “dominance” and “sequencing/SGTF.”

Studies were conducted in 10 different countries and mainly used national electronic registries or claimed data from the general population for laboratory, immunization, and patient characteristics. Only three studies investigated VE for specific populations such as healthcare workers, veterans, or patients under hemodialysis therapy. The minimum age of included study participants was 12 years (if reported). However, none of the studies provided subgroup data for children or adolescents.

Twenty-two studies reported VE estimates for full primary immunization (as defined per study) and 23 for a booster dose (additional dose after full primary immunization). One of the latter studies compared the effect of a second booster dose (in the study referring to a fourth dose of a mRNA vaccine) with a single booster dose (third dose of a mRNA vaccine). VE estimates for several time points after full primary immunization have been reported by 11 studies, and 8 studies reported VE data for several time points after booster immunization.

Most studies investigated VE of mRNA-based vaccines (13 on Comirnaty and 9 on Spikevax), seven studies reported VE for vector-based vaccines (four on Vaxzevria and three on COVID-19 vaccines from Janssen), and seven studies did not differentiate VE per vaccine. None of the studies provided data for Nuvaxovid.

### Prevention of infection with SARS-CoV-2 Omicron variant (without differentiation between asymptomatic or symptomatic cases)

**Figure 2:**
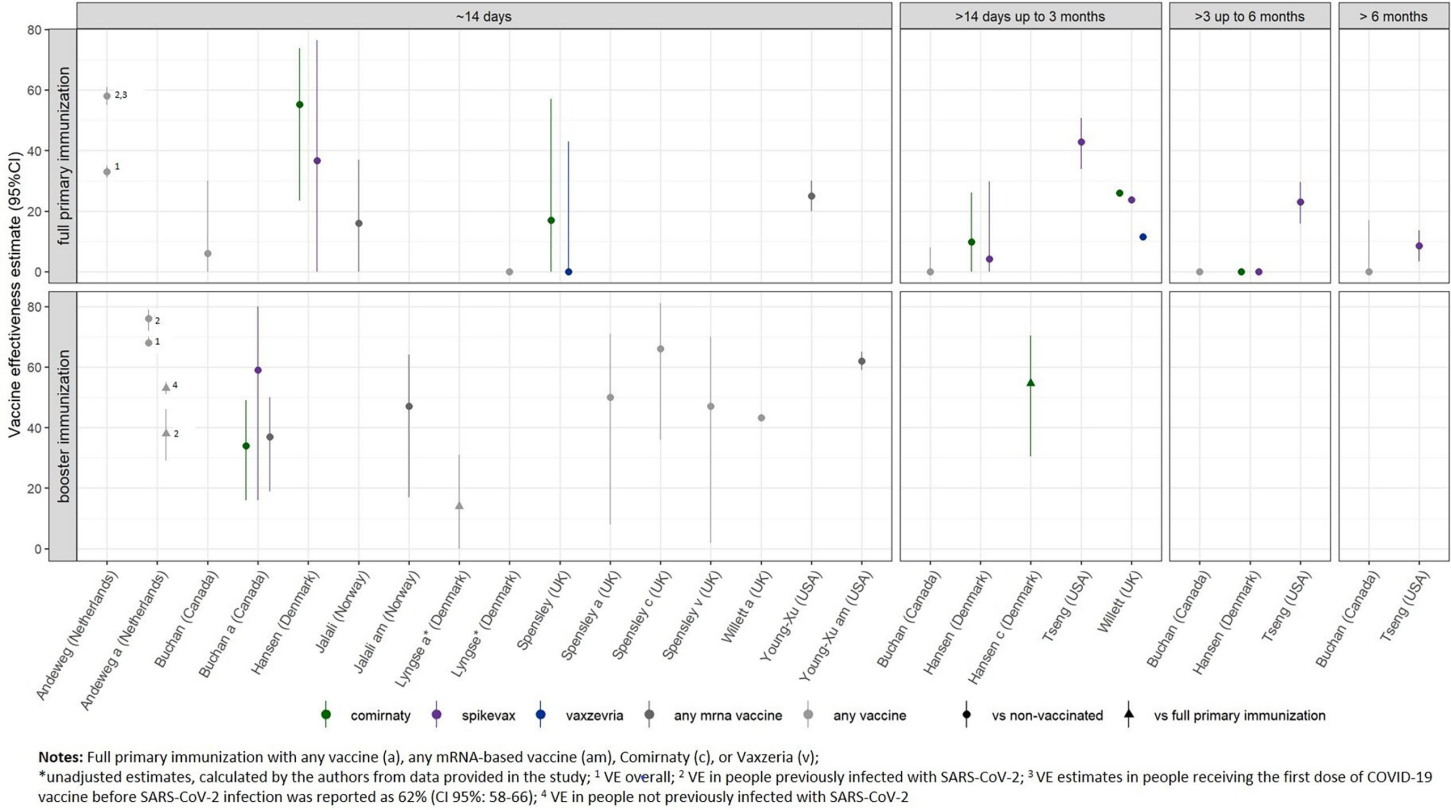
Vaccine effectiveness estimates against SARS-CoV-2 infection (any type) of the Omicron variant after full primary immunization and booster dose, as reported in the study for the defined time strata after immunization. For booster immunization the COVID-19 vaccine used for primary immunization is indicated.

Twelve studies (including between 1,220 and 2.2 million participants) reported the effectiveness of COVID-19 vaccines in preventing infection of any type with SARS-CoV-2 Omicron variant (without differentiation between asymptomatic or symptomatic cases). After full primary immunization, VE across all studies ranged between 0% and 62% at “≈14 days” post-vaccination, compared to no vaccination. For the time periods of “>14 days up to 3 months,” “>3 months up to 6 months,” and “>6 months” after vaccination, VE ranges, assessed across all reported VE estimates, were 4.2%–42.8%, 0%–23%, and 0%–8.6%, respectively (**Table 1**, **Figure 2**). The data from the two studies reporting VE for at least two time points show a decline of VE between >14 days to up to 6 months by 16%–34% after vaccination with mRNA-based vaccines (**Figure 2**) (3, 4). As there was no VE data over time identified for vector-based vaccines, unspecified vaccines, or heterologous schedules, the respective VE decline could not be assessed.

After booster vaccination, VE estimates at “≈14 days” post-vaccination across all studies ranged between 34% and 76%, compared to no vaccination, and between 14% and 53%, compared to full primary immunization (9, 11). Follow-up data were insufficient to evaluate the waning of immunity after booster vaccination.

The study investigating the VE of four vs. three doses of mRNA-based vaccines reported a VE estimate of 47% (95% CI, 44–47) against Omicron infection at 12 or more days after the fourth dose (29). VE ranges against infection with the Delta variant for the different time points are provided in **Supplementary Material 1**, Part 5.

To our knowledge, no study provided VE estimates for the direct comparison of different vaccine formulations. When comparing provided estimates across studies, the VE of mRNA-based vaccines is suggested to be higher than that of vector-based vaccines [16% (0–37) to 55.2% (23.5–73.7), respectively, −4% (−97−43) to 11.4 (NR to NR)] at ...≈14 days after full primary immunization. For later time points and booster immunization, VE could not be compared as data for vector-based vaccines were not available.

**Figure 3:**
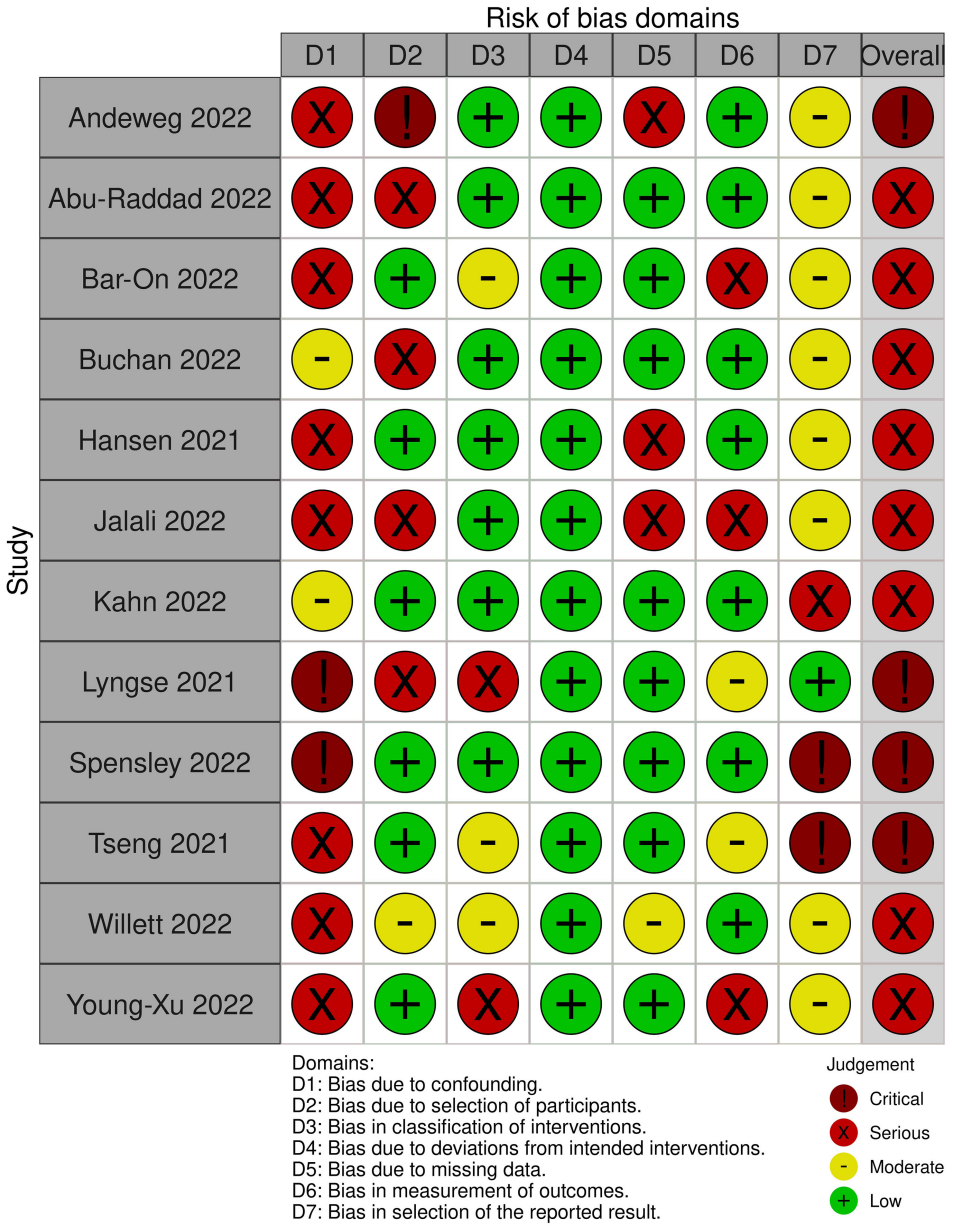
Risk of Bias assessments for the outcome "SARS-CoV-2 infection (any type)".

The risk of bias was serious to critical for all assessed studies (see **Figure 3**). A key concern was no or insufficient adjustment for confounders.

### Prevention of symptomatic COVID-19

**Figure 4:**
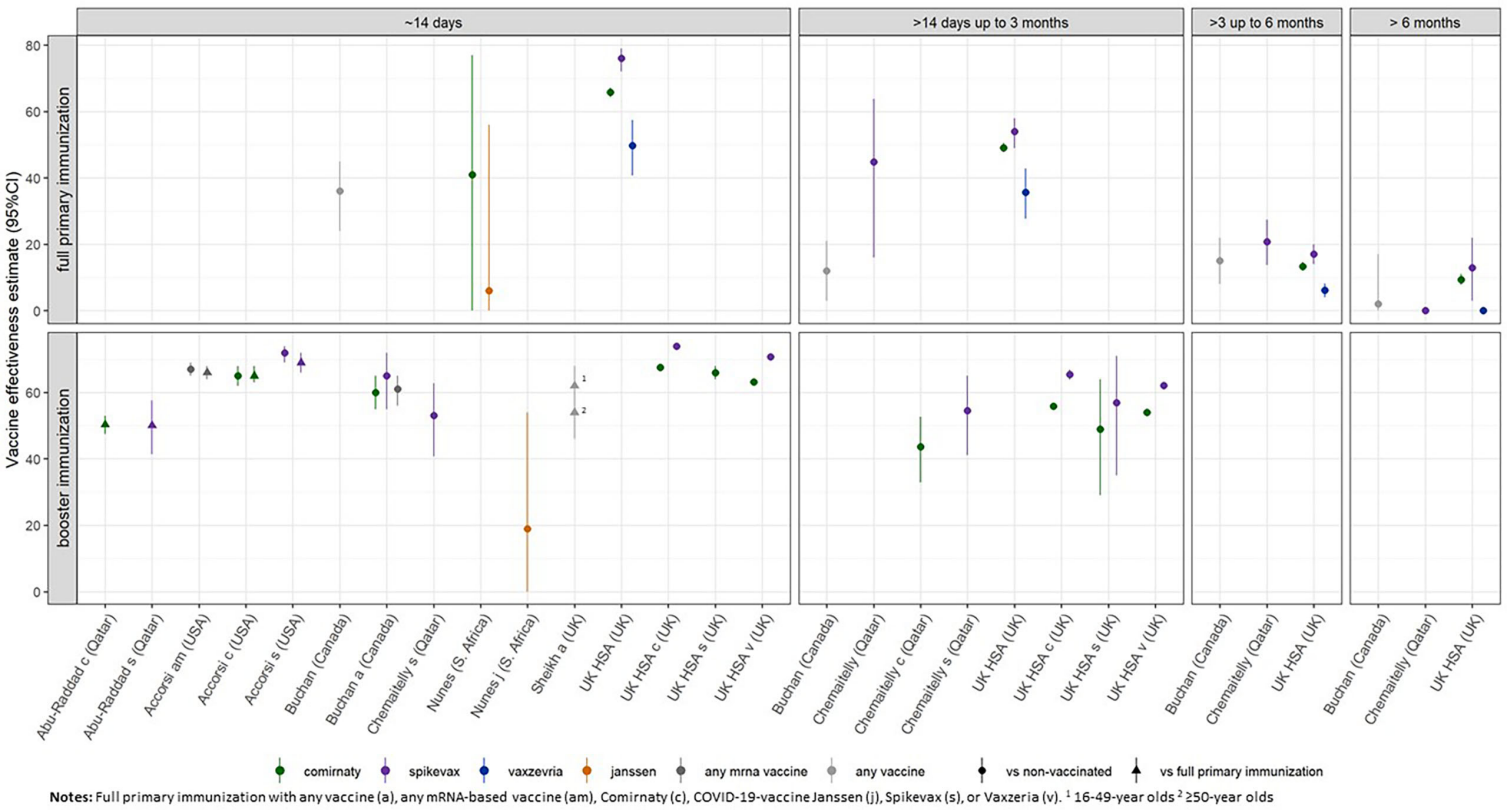
Vaccine effectiveness estimates against symptomatic COVID-19 due to SARS-CoV-2 infection of the Omicron variant after full primary immunization and booster dose, as reported in the study for the defined time strata after immunization. For booster immunization the COVID-19 vaccine used for primary immunization is indicated.

Seven studies, including between 430 and 2.2 million participants, estimated the effectiveness of COVID-19 vaccines in preventing symptomatic infection with the SARS-CoV-2 Omicron variant (**Supplementary Material 1**, Part 4). Compared to unvaccinated individuals, VE at ∼14 days after full primary immunization summarized from all studies reporting on this time point ranged between 6% and 76%. For the time period of “>14 days up to 3 months,” “>3 months up to 6 months,” and “>6 months,” VE ranges were 12%–54%, 6.1%–20.8%, and 0%–13%, respectively. VE estimates from studies that report data for at least two time points suggest a decrease in protective immunity against symptomatic COVID-19 between 45% and 63% for mRNA-based vaccine recipients and 50% for vector-based vaccine recipients over the time period of >14 days up to >6 months after full primary immunization (**Figure 4**) (13, 14).

After booster vaccination, VE against symptomatic infection ranged between 19% and 73.9% at “≈14 days” post-vaccination, compared to no vaccination, and between 50% and 68%, compared to full primary immunization (12, 16). For the time point of “>14 days up to 3 months,” estimates were only reported for the comparison with no vaccination and ranged between 43.7% and 65.4%. No study reported VE for later observation periods. The two studies that provide data for both these time periods show a 9%–28% decrease in VE after booster vaccination with an mRNA-based vaccine and between 32% and 34% after heterologous vaccination schemes (**Figure 4**) (13, 14). However, 95% confidence intervals between observation periods were partially overlapping.

To our knowledge, no study provided VE estimates directly comparing different vaccine formulations. Of the included studies, only a few studies investigated the VE of vector-based vaccines against symptomatic diseases. Based on these, VE against symptomatic COVID-19 compared across studies is suggested to be higher for mRNA-based vaccines at the time points of “≈14 days,” “>14 days up to 3 months,” and “>3 months up to 6 months” after full primary immunization and at “≈14 days” after booster immunization (**Table 1**).

**Figure 5:**
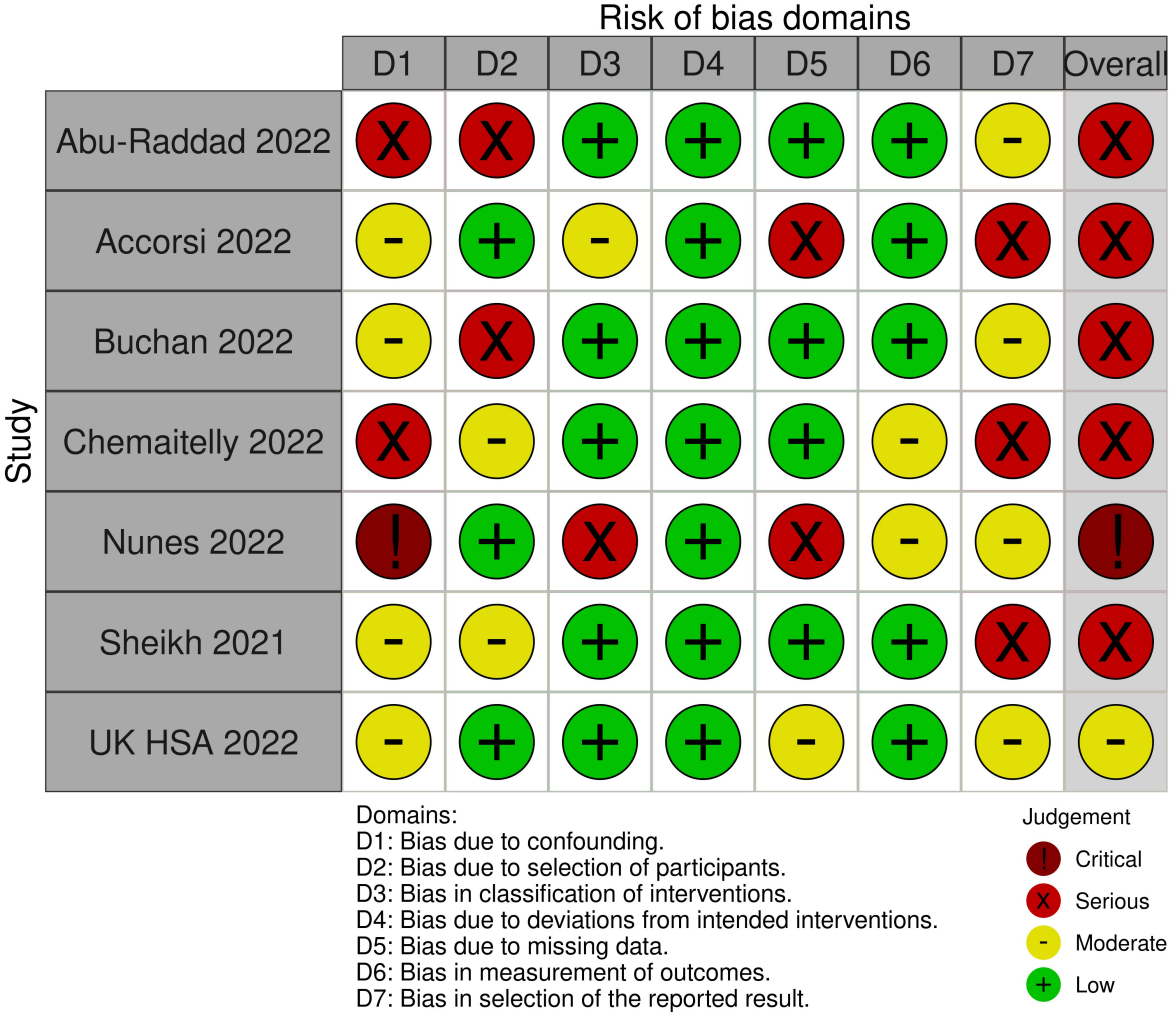
Risk of Bias assessments for the outcome "symptomatic COVID-19" due to SARS-CoV-2 infection of the Omicron variant.

The risk of bias was serious to critical for all but one of the assessed studies (see **Figure 5**). The remaining study was rated to have a moderate risk. All factors considered relevant for confounding were taken into account; however, residual confounding could not be ruled out.

### Prevention of severe COVID-19 (hospitalization, ICU admission, or death)

**Figure 6:**
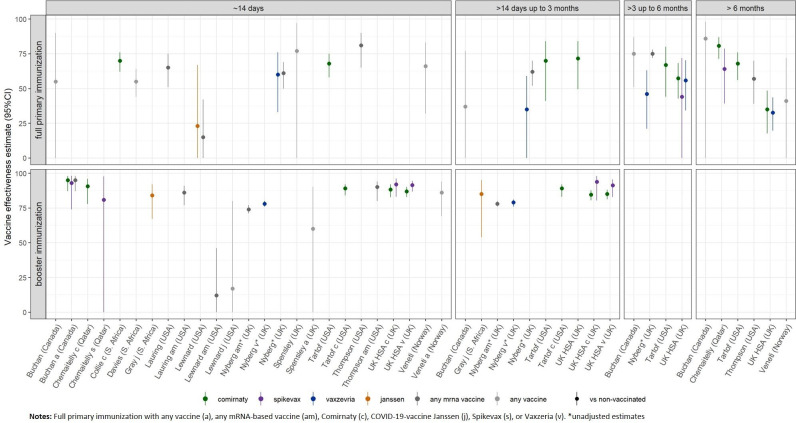
Vaccine effectiveness estimates against severe COVID-19 (incl. hospitalization, ICU admission or death) due to SARS-CoV-2 infection of the Omicron variant after full primary immunization and booster dose, as reported in the study for the defined time strata after immunization. For booster immunization the COVID-19 vaccine used for primary immunization is indicated.

VE against severe COVID-19 was assessed in 17 studies including 1,220 to 2.2 million participants (**Supplementary Material 1**, Part 4). VE estimates in full primary vaccinated compared to unvaccinated individuals ranged from 3% to 84% at “≈14 days” post-vaccination, between 21% and 95% at “>14 days up to 3 months,” between 0% and 91% at “>3 months up to 6 months,” and between 32.7% and 86% at “>6 months” after vaccination. Studies reporting VE for at least two time points indicate a decline by up to 40% for mRNA-based vaccines and 15%–67% for vector-based vaccines between 14 days and ≥6 months after vaccination (13, 22). However, 95% confidence intervals were wide and overlapping across time points. One study reported no difference in the first and last time point estimate (20), and another study reported a small non-significant increase [VE at 30–180 days: 73.7% (95% CI, 46.8–87); VE at ≥210 days: 80.7% (95% CI, 71.3–87) (14)] (see **Figure 6**, **Supplementary Material 2**).

**Figure 7:**
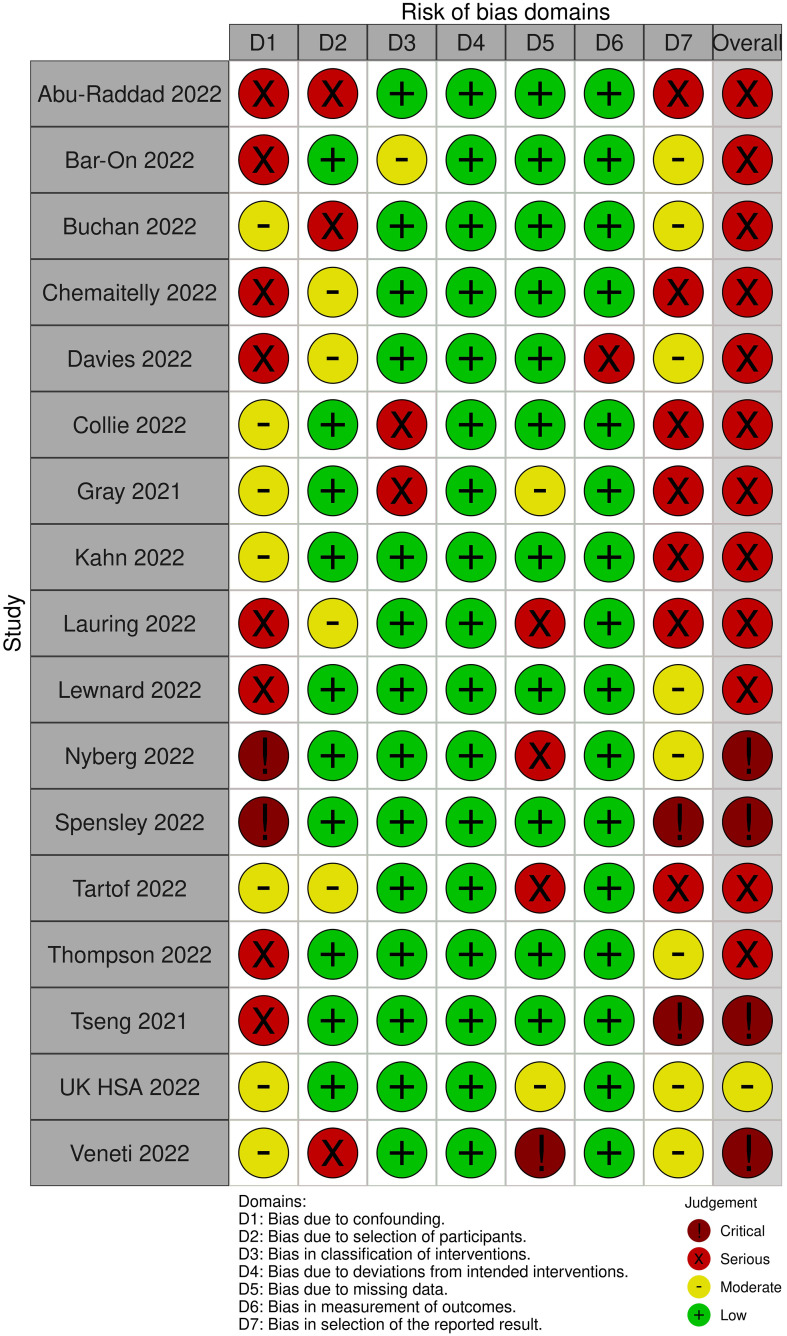
Risk of bias assessments for the outcome "severe COVID-19" due to SARS-CoV-2 infection of the Omicron variant.

For booster vaccinated compared to unvaccinated individuals, VE ranged from 12% to 100% at “≈14 days” post-vaccination and between 78% and 93.7% at “>14 days up to 3 months”. According to three studies, VE after booster vaccination remained stable over this time period (14, 20, 22), irrespective of the vaccine or scheme used (mRNA-, vector-based, or heterologous vaccination). Only one study showed a decline of VE of approximately 12% over the respective time period after mRNA-based vaccination (13). VE data was not available for later time periods.

For the comparison against full primary immunization, we calculated the VE of booster vaccination against severe disease based on data from one study (12) that only reported data for 7 days after booster vaccination [VE: 100% (95% CI, 71.4–100)].

When comparing VE data of different vaccine formulations against severe COVID-19 across studies, no substantial differences were identified. At ∼14 days after full primary immunization, the VE of mRNA-based vaccines was slightly lower than the VE of vector-based vaccines; at the time points of “>3 months to 6 months” and “>6 months” after full primary immunization, the VE of mRNA-based vaccines was slightly higher than VE of vector-based vaccines at respective time points. VE estimates after booster immunization were similar for both vaccine formulations at the time points of “≈14 days” and “>14 days up to 3 months.” To our knowledge, no study provided VE estimates directly comparing different vaccine formulations.

The study assessing the effect of a fourth vs. a third mRNA-based vaccine dose reported VE against severe disease of 75% (95% CI, 57–86) at ≥12 days after additional booster vaccination (29). Data to assess the duration of protection were not available.

In all but one studies, the risk of bias was serious to critical (see **Figure 7**). The remaining study was rated to have a moderate risk due to the potential for residual confounding.

### Effectiveness against Omicron variant, compared to Delta variant

Twenty-one studies reported VE estimates both against infections with the Omicron and with the Delta variant (**Supplementary Material 1**, Part 4).

In addition to our PICO question, we included eight studies that estimated the risk reduction of infections with the Omicron variant compared to those with the Delta variant (**Supplementary Material 1**, Part 6). Risk ratios from these studies suggest that the risk for any type of SARS-CoV-2 infection and symptomatic COVID-19 despite vaccination is higher for the Omicron variant than for the Delta variant. Instead, the risk of severe disease is lower in vaccinated people infected with the Omicron variant compared to those vaccinated and infected with the Delta variant (**Supplementary Material 1**, Part 7).

### Publication bias

Potential publication bias could not be explored through statistical testing and generating funnel plots, as none of the comparisons/outcomes/time points involved sufficient studies.

### GRADE

Overall, the GRADE certainty of the evidence is very low for all outcomes due to the underlying study limitations and serious heterogeneity.

## Discussion

This third update of our LSR provides evidence on VE and duration of protection of EU-approved COVID-19 vaccines against any type of infection, symptomatic infection, and COVID-19-associated severe disease (i.e., hospitalization, ICU admission, or death) caused by the SARS-CoV-2 Omicron variant.

Although evidence is uncertain about the exact level of protection against all investigated outcomes, both after full primary and after booster vaccination, data suggest that the VE was higher after booster immunization when compared to full primary immunization. Results suggest a rapid decline in vaccine-induced protection after the completion of the full primary vaccination series. The effect is profound for infections of any type but less pronounced for severe diseases. This is in line with the findings of a recently published meta-regression analysis on pre-Omicron variants (32). VE could be restored to high levels of protection by the booster dose, although the first follow-up data also suggest a waning effect (13, 14, 21). However, VE against severe disease caused by Omicron remained high for at least 3 months post-booster immunization (no data for longer follow-up available). Compared to VE against the Delta variant, vaccines were less effective for all reported outcomes.

In light of the more transmissible Omicron variant and the advancing immunization campaigns across countries, evidence of the need and the timing of booster vaccination is of increasing public health interest. Thus, we decided to adapt our PICO questions and inclusion criteria and considered also studies that compare booster-vaccinated individuals with primary-vaccinated ones. The effect of waning immunity was assessed by stratifying VE data into multiple observation periods.

As included studies were highly heterogeneous (e.g., in terms of analyzed time points after vaccination, study population, applied vaccine schedules, if reported at all) and had a serious to critical risk of bias, we decided not to perform meta-analyses but provide ranges of reported VE estimates across studies to increase transparency and prevent misinterpretation. Due to the heterogeneity of the studies, the VE ranges provided for all of the outcomes are very wide, even when disaggregated by vaccine type. This is especially true for the VE estimates comparing full primary immunization with unvaccinated, which may mainly be the result of differences among the study populations, who were not accounted for in the adjustment measures, e.g., level of naturally acquired immunity, underlying comorbidities, and/or socio-economic parameters. The studies adjusted for very different confounders, but none adjusted for all variables the study team considered relevant. For any type of infection including symptomatic COVID-19, the study team considers age, sex, region, time, underlying comorbidities, previous SARS-CoV-2 infection, occupation, previous influenza vaccination, nationality, ethnicity, and socioeconomic status important confounders, whereas for severe COVID-19, age, sex, time, underlying comorbidities, previous COVID-19 treatment, duration since symptom onset, region, and previous SARS-CoV-2 infection are considered important confounders.

Recent *in vitro* data suggest that substrains of the Omicron variant “escape” the immune response induced by immunization in different forms, possibly leading to different VE against different substrains of the Omicron variant. In mid-January 2022, an increase in substrain BA.2 was observed in most countries. Of the included studies, only one reported VE data stratified by underlying substrains BA.1 and BA.2 of the Omicron variant. These data suggest that VE against these substrains did not differ substantially [VE after 25+ weeks after two doses: BA.1, 9% (7–10%); BA.2, 13% (−26%–40%); VE after 2 weeks after booster immunization: BA.1, 63% (63%–64%); BA.2, 70% (58%–79%) (33)]. More recent studies confirm this finding (9, 34), making it unlikely that the wide VE ranges are due to the different substrains circulating at the time when the included studies were conducted.

A standardized assessment for time points of VE evaluation as suggested by the WHO (35) and for the confounders considered would facilitate the comparison of evidence across studies and allow statistical synthesis of the evidence.

Most studies included here used the test-negative design. This study design was initially introduced to estimate VE against seasonal influenza and thought to control for differences in seeking medical care (36). In the COVID-19 era, the test-negative studies might be prone to bias caused by specific testing strategies or behaviors at the study location. However, those are mainly not reported in the studies, making it difficult to interpret reported estimates when individuals are not tested due to underlying symptoms. Most studies did not indicate the vaccination schedule applied for full primary immunization or the booster dose. For immunocompromised people, a three-dose primary vaccination schedule is recommended by the WHO Strategic Advisory Group of Experts on Immunization to improve immune response. As this recommendation is implemented in many countries^1^, it is possible that VE estimates for a booster schedule include those that received third doses within an “optimized” schedule for immunocompromised people.

This third update provides a comprehensive overview of the currently available evidence on VE against infection with the SARS-CoV-2 Omicron variant. We provide an in-depth analysis of the VE estimates extracted from the included studies that were identified following a pre-registered protocol. We conducted a thorough risk of bias assessment of the studies and evaluated the certainty of evidence using the GRADE approach.

Due to the highly dynamic publication landscape in this field, additional studies have been published since our last search that are not captured by this analysis. We are aware of at least two studies reporting on adolescents, which were published after our data cut (37, 38). As in adults, lower VE against infections and severe disease was observed for the Omicron variant when compared to the Delta variant. However, data on the duration of protection are contradicting. While one study reported a decrease in VE against hospitalizations by 6%–19% at more than 5 months after full primary immunization (37), the second study did not identify a decrease in VE against any infection but was based only on few events (38). It should be noted that most studies (21/26) included in this systematic review have not yet undergone the peer-review process. This should be taken into account when interpreting the reported results. In addition to studies not included, as they were published after our final search date, we noticed that some studies updated data on pre-print servers after initial publication including data on longer follow-up periods. We therefore cannot exclude that the authors revised the available pre-print versions including additional data after we completed data extraction for this update. Furthermore, we cannot exclude a risk of potential bias as real-life observations are based on retrospective analyses that are not systematically registered. Thus, intent and possibility of publication might depend on observed results. However, due to the increase in publications on pre-print servers, the risk of publication bias is probably low.

## Conclusion

Current evidence suggests that the effectiveness of COVID-19 vaccines licensed in the EU is low after full primary vaccination and improved after booster vaccination in preventing infections with the SARS-CoV-2 Omicron variant. For both full primary vaccination and booster immunization, it is characterized by a rapid decrease over time. VE against severe courses of COVID-19 remains generally high.

The studies included in this update were very heterogeneous. Therefore, the pooling of estimates was not appropriate. To allow statistical synthesis of effects, studies need to be comparable for clinical and meta-epidemiological aspects. Thus, certain standards for VE studies, as suggested by WHO, are useful to better inform vaccination guidelines and reliably assess the public health impact of vaccination campaigns.

## Data availability statement

The original contributions presented in the study are included in the article/**Supplementary Material**. Further inquiries can be directed to the corresponding author.

## Author contributions

WKS was first and second reviewer, responsible for data extraction, performed bias assessment and drafted the manuscript. VP was first and second reviewer, responsible for data extraction, performed bias assessment, conducted GRADE assessment and contributed to the manuscript. AP was first and second reviewer, responsible for data extraction, performed bias assessment and contributed to the manuscript. MB was first reviewer, responsible for data extraction, performed bias assessment and contributed to the manuscript. MW, SK, LSD, and BG were first reviewers and contributed to the manuscript. MT provided input into the interpretation of the results and developed forest plots. SVB and JK contributed to the manuscript. OW held general oversight of the conducted work and revised the manuscript. TH conceived the study and contributed to the manuscript. All authors contributed to the interpretation of the data and provided important intellectual content to the manuscript. All authors contributed to the article and approved the submitted version.

## Acknowledgments

We sincerely thank the authors Andrews et al. and Kahn et al. for providing us with additional data.

## Conflict of interest

The authors declare that the research was conducted in the absence of any commercial or financial relationships that could be construed as a potential conflict of interest.

## Publisher’s note

All claims expressed in this article are solely those of the authors and do not necessarily represent those of their affiliated organizations, or those of the publisher, the editors and the reviewers. Any product that may be evaluated in this article, or claim that may be made by its manufacturer, is not guaranteed or endorsed by the publisher.

## Footnotes

1 An additional dose is recommended for immunocompromised people in all eight countries where included studies that reported VE estimates on booster immunization were conducted (Canada, Denmark, Netherlands, Norway, Qatar, South Africa, United Kingdom, and United States of America).
